# Skill enactment and knowledge acquisition among community users of digital mental health interventions: qualitative study with thematic analysis

**DOI:** 10.1186/s12888-024-05953-3

**Published:** 2024-08-01

**Authors:** Hayley M. Jackson, Philip J. Batterham, Jeneva L. Ohan, Alison L. Calear, Louise M. Farrer

**Affiliations:** 1 Centre for Mental Health Research, National Centre for Epidemiology and Population Health, The Australian National University, Canberra, Australia; 2 School of Psychological Science, The University of Western Australia, Crawley, WA Australia

**Keywords:** Internet, Computer-assisted therapy, Engagement, Skill enactment, Qualitative

## Abstract

**Background:**

The acquisition of knowledge and use of skills from digital mental health interventions (DMHIs) are considered important for effectiveness. However, our understanding of user experiences implementing skills learned from these interventions is limited, particularly outside of research trials. This qualitative study aimed to investigate how community users learn and apply knowledge and skills from DMHIs based on cognitive behavioural therapy (CBT) in daily life. The study also examined factors influencing the selection and use of skills and explored perceived changes in mental health resulting from the intervention.

**Methods:**

Thirteen adults aged 26 to 66 years (10 females) were recruited using social media advertising and participated in semi-structured interviews by telephone or videoconference. All participants were living in Australia and had used a digital CBT program within the past 3 months. Interviews lasted on average 45 min. Transcripts were analysed using theoretical thematic analysis.

**Results:**

Participants demonstrated high levels of program engagement. Findings were organised into three topics with six major themes. Participants reported that their chosen intervention reinforced existing knowledge and fostered new skills and insights (Topic 1, Theme 1: knowledge consolidation). Most described actively applying skills (Topic 1, Theme 2: active approach to skill enactment), although the extent of learning and range of skills enacted varied across participants. Influences on skill selection included the perceived relevance of intervention strategies to the user’s needs and personal characteristics (Topic 2, Theme 1: relevance of intervention strategies), as well as the perceived or experienced effectiveness of those strategies (Topic 2, Theme 2: perceived and experienced benefit). Challenges to ongoing skill enactment included time scarcity, prioritisation difficulties, and lack of motivation (Topic 2, Theme 3: navigating time constraints and low motivation). Improvements in mental health were generally modest and attributed mainly to participants’ proactive efforts (Topic 3, Theme 1: perceived changes).

**Conclusions:**

DMHIs may reinforce existing understanding of psychotherapeutic strategies, offer new knowledge, and encourage the application of skills in everyday life among community users who actively engage with these interventions. Future research should prioritise personalising DMHIs and investigating methods to optimise the acquisition, retention, and sustained application of knowledge and skills.

**Supplementary Information:**

The online version contains supplementary material available at 10.1186/s12888-024-05953-3.

## Background

Approximately one in five adults meet the diagnostic criteria for a mental health disorder each year [1]. Many of these individuals do not receive timely or appropriate care [2]. Digital mental health interventions (DMHIs) have emerged as potential solutions to improve access to evidence-based services [3]. These interventions are delivered through internet websites or smartphone apps and can be used with or without human support [4]. Meta-analyses have demonstrated the effectiveness of DMHIs in reducing symptoms associated with a range of common mental health problems such as depression and anxiety disorders [5, 6]. Interventions based on cognitive behavioural therapy (CBT) have the most robust evidence supporting their effectiveness [7]. However, not all individuals who use these interventions will experience benefits [8]. This is due in part to levels of user engagement [9], which have frequently been found to be suboptimal in research settings [10] and tend to be even lower among free-range users in the community [11].

Existing literature suggests that user engagement is influenced by several factors, including the perceived relevance of an intervention to user needs, levels of personalisation and interactivity, and the availability of external support or guidance [12]. Individual and demographic differences have also been found to influence engagement, with higher engagement generally observed among females, younger people, those with less severe depression at baseline, and those with more positive beliefs about DMHIs [12]. A limitation of this evidence is that most previous studies have considered these factors in relation to program usage indicators of engagement, such as the number of modules or exercises completed [13]. Recommendations in user engagement research increasingly highlight the need to consider the application of strategies and techniques learned from an intervention in everyday life (i.e., skill enactment) as an additional dimension of engagement [14, 15]. Skill enactment is thought to play an important role in producing treatment effects following CBT and other digital interventions that involve skills training [14, 16]. These programs aim to facilitate improvements by providing users with cognitive and behavioural skills that can be used to cope with and manage symptoms [17]. However, there is currently inconclusive evidence regarding the role of skill enactment in symptom reduction during and after participation in digital CBT. Although some studies have demonstrated improvements in skill enactment alongside mental health outcomes [18, 19], others have failed to show such changes [20, 21]. Additionally, some studies report a non-significant association between skill enactment and post-intervention mental health outcomes [22]. These differences may be attributed to various factors, including differences in intervention length and duration, the presence or absence of tailoring and other engagement features, as well as the availability of guidance [23]. Different skills may also be relevant across different symptom severities and user groups [24]. However, few studies have investigated the processes or factors that underpin successful skill enactment.

One factor that is thought to be necessary for effective skill enactment is the acquisition of knowledge about mental disorders and strategies to address symptoms (referred to as knowledge acquisition hereafter). This is particularly pertinent in self-help programs, where independent learning and self-directed use of intervention materials are emphasised. Psychoeducation is a common component of CBT programs and is typically incorporated with the aim to improve knowledge and understanding of mental disorders, their symptoms, and the underlying cognitive and behavioural patterns contributing to distress. Psychoeducation has been shown to produce small improvements in health outcomes, even in the absence of therapeutic strategies [25]. There is also evidence that digital CBT interventions can produce improvements in explicit recall of CBT principles [14]. However, existing evidence suggests that neither CBT-specific knowledge gain nor improvements in general mental health literacy are related to treatment outcomes following digital CBT [14, 26], and limited research has investigated how improvements in knowledge are related to skill enactment.

Inconclusive evidence regarding the role of skill enactment and knowledge acquisition may be partly attributed to the limitations of quantitative measures in capturing the complexities of these constructs. Qualitative research enables a deeper exploration of the experiences, perspectives, and contextual factors influencing skill enactment and knowledge acquisition. This information is important for driving innovation in the design and implementation of digital interventions. Two previous studies investigated user experiences of learning and applying knowledge gained during digital CBT interventions using qualitative interview methods [27, 28]. Both observed a distinction between participants who actively engage with and apply knowledge and skills versus those who adopt a more passive or reactive approach. Both studies also focused on user experiences following participation in randomised controlled trials. These settings differ from community-based contexts in ways that may have implications for engagement (e.g., use of explicit assessment protocols and researcher contact). Moreover, interviews took place after an extended time frame, potentially making it difficult for participants to accurately recall initial treatment experiences. The studies also lacked a thorough exploration of factors impacting on initial and ongoing skill enactment or on the use of specific strategies. Addressing these gaps can enhance our understanding of engagement with DMHIs in real-world contexts and guide future intervention design to better account for user experiences and perspectives.

This study used a critical realist paradigm [29] to explore community users’ experiences and perspectives of digital CBT programs within 3 months of intervention use, with a focus on skill enactment and knowledge acquisition. Critical realism asserts the existence of an objective reality, while acknowledging that our understanding of this reality is fallible and influenced by social, cultural, and historical contexts [30]. The approach has been applied across various domains of health research, including digital mental health [31–33], aiming to identify and explain complex health phenomena [34]. In this study, we sought to investigate: (1) how community users learn and apply information and skills from DMHIs in daily life, (2) the factors influencing their use of different therapeutic strategies, and (3) perceived changes in mental health and wellbeing resulting from the intervention.

## Methods

### Overview

The present study reports the results of semi-structured interviews conducted with 13 Australian adults who had used a CBT-based DMHI within the past 3 months. The research team approached the project with an interest in understanding experiences of learning and skill enactment among community users of DMHIs in order to inform future research and intervention design. The first author (HMJ) led the study and conducted and analysed the interviews. HMJ is a female doctoral researcher with training in psychology whose PhD research focuses on engagement with DMHIs. She has previous experience conducting interviews and analysing qualitative data (see [35]). She was interested in new insights and perspectives from the participants, acknowledging the potential impact of her own worldview on the interpretation of findings. Ongoing supervision was provided by the senior author (LMF), a psychologist and researcher in digital mental health. The study is reported according to the COnsolidated criteria for REporting Qualitative Research (COREQ) checklist [36] (refer to Appendix 1).

### Recruitment and screening procedure

Participants were purposively sampled into the study over a period of approximately three weeks, from 3 October to 20 October 2023, using targeted social media advertising on Facebook and Instagram. Advertisements targeted Australian adults who were identified as having an interest in mental health and wellbeing based on keywords and community interests associated with their social media profile. Individuals who clicked on the study advertisement were taken to an online Qualtrics survey containing study information and a question to obtain informed and voluntary consent. Respondents were eligible for inclusion if they were aged 18 years or older, currently residing in Australia, and had used a CBT-based DMHI (i.e., online program or smartphone app) within the past 3 months. To confirm eligibility, participants were asked to select from a list of interventions (derived from e-Mental Health in Practice [37] and Beacon [38]) or state the name of the intervention they used most often within the past 3 months. Fictitious program names were listed alongside legitimate programs in an effort to minimise data quality issues. Where participants stated the name of the intervention they used (rather than selecting from the listed interventions), the program was manually screened by the first and senior authors to confirm that it was based on CBT before inviting the participant to interview. CBT-based programs were classified as interventions that included cognitive restructuring as a core component. Respondents who selected a fictitious program (*n* = 1) or who had not participated in a CBT-based program (*n* = 5) were excluded and provided with help-seeking resources.

### Participants

Table 1 presents the characteristics of study participants. Sixteen people were eligible and consented to participate in the study. Of these, three were subsequently unable to be contacted, either to schedule or complete the interview. Therefore, 13 participants aged 26 to 66 years (*M* = 39.69 years, *SD* = 11.09) participated in the study. Of these, 10 (77%) identified as female and three as male. Most participants had a university degree, and the majority were living in Victoria or New South Wales. Participants reported various mental health concerns, including depression or low mood, anxiety, sleep problems, and a general dissatisfaction with their current mental health. For some participants, these problems were self-described as severe or protracted, and a couple described experiencing significant trauma or stressful life events that contributed to the development of these issues. All participants had used a CBT-based DMHI within the past three months, and three were still working with the program at the time of the interview.

**Table 1:** Characteristics of the participants

ID^a^	Age^b^	Gender	Ethnicity^c^	State	Education^b^	Program(s)	Presence of guidance	Primary concern^e^
F1	25–34	Female	South Asian	Victoria	Undergraduate degree	THIS WAY UP Social Anxiety Program; Health Anxiety Program^d^	Unguided	Health anxiety, social anxiety
M2	35–55	Male	Chinese	New South Wales	Postgraduate degree	HeadGear	Unguided	Sleep issues, generalised mental health concerns
M3	≥ 65	Male	Northern European/ Caucasian/ Anglo	New South Wales	Undergraduate degree	THIS WAY UP Health Anxiety Program	Unguided	Health anxiety
F4	25–34	Female	None	Victoria	Vocational education	THIS WAY UP Generalised Anxiety Program; Depression Program^d^	Unguided	Anxiety
F5	25–34	Female	Indian	Victoria	Undergraduate degree	MumMoodBooster	Coach supported	Depression
F6	35–44	Female	Australian	New South Wales	Secondary education	THIS WAY UP Mixed Anxiety and Depression Program	Unguided	Depression, Anxiety
M7	25–34	Male	Indian	Victoria	Postgraduate degree	MoodMission, CBT Companion, What’s Up?^d^	Unguided	Low mood
F8	35–44	Female	Asian	New South Wales	Undergraduate degree	THIS WAY UP Insomnia Program	Unguided	Sleep issues
F9	25–34	Female	Caucasian/ New Zealander	New South Wales	Vocational education	MindSpot Wellbeing Course	Unguided	Multiple mental health concerns, low quality of life
F10	35–44	Female	South Asian	Victoria	Postgraduate degree	MumMoodBooster	Coach supported	Generalised mental health concerns
F11	45–54	Female	New Zealander	Victoria	Undergraduate degree	MindSpot Wellbeing Course	Therapist supported	Generalised mental health concerns
F12	45–54	Female	White Australian	Queensland	Undergraduate degree	moodgym	Unguided	Anxiety, low mood
F13	25–34	Female	Asian	Victoria	Undergraduate degree	THIS WAY UP Depression Program	Unguided	Depression

^a^Participants are identified by their gender and a coded number

^b^Broad classifications are reported to hide the exact age and qualifications of the participants

^c^Self-identified ethnicity is reported

^d^Participant used two or more eligible interventions

^e^Self-identified concerns are reported

### Data collection

Semi-structured interviews were conducted to capture variability in the experiences of DMHIs among community users, while covering the key topics of interest with all participants. Participants were given the choice of participating in an interview via telephone or Zoom, as research suggests that offering multiple interview formats can improve participant recruitment and data quality [39]. Eight interviews were conducted by telephone, with the remaining five conducted on Zoom. The interviews lasted between 21 and 65 min, with an average length of 46.2 (*SD* = 14.9) minutes. Participants were informed that the interviewer was a PhD student researching user engagement with DMHIs prior to participating. They were advised to choose a quiet space away from others to maintain confidentiality and encourage open discussion. The interview questions were developed by the research team to explore the following topic areas: (1) how participants learned and applied information and skills from the DMHI in their daily lives; (2) factors influencing their selection and use of therapeutic techniques; and (3) perceived changes in their mental health or wellbeing and their thoughts regarding what may have brought about these changes. The full list of interview questions is provided in Appendix 2. Example questions include: “Did you learn anything new from the program/app?” and “Have you continued to use anything from the program/app in your everyday life? If so, in what way?”. No substantive changes were made to the interview guide during the study. Prompts and follow-up questions were used to encourage participants to clarify or elaborate on their responses, and to delve deeper into specific areas of interest as they arose during conversation. Demographic information was collected at the beginning of each interview, and usage levels were determined by asking participants how much of the program or app they had completed.

We used an information power approach to guide sample size decisions [40], considering the study aims, sample characteristics, quality of participant interactions, and analytic method during both study design and data collection. Data were reviewed during data collection, and the number of participants was adjusted downwards from 15 (estimated during study design) to 13 due to the high quality of participant dialogue and high relevance of data to the research questions. This sample size was deemed sufficient to yield rich and experientially diverse data [41]. The interviewer had no prior relationship with the participants, and repeat interviews were not conducted. Field notes were recorded after each interview to document important observations and contextual information.

### Transcription and analysis

Data were managed using Quirkos (desktop version). Interviews were audio-recorded and transcribed verbatim using Otter.ai, an automated speech-to-text transcription application. Transcripts were corrected and de-identified by the first author (HMJ) while listening to the audio-recordings. All participants were offered the opportunity to review their transcript; de-identified copies were provided to six participants, and four returned their transcript following review. No changes were suggested. For the remaining two participants, consent to use their transcript was assumed when no communication was received within two weeks, per the ethics protocol. This process was communicated to participants through the study information sheet, on conclusion of the interview, and in email correspondence containing the corrected transcript. Transcripts were analysed using theoretical thematic analysis [42]. This approach was chosen as it provides a systematic method to identify repeated patterns of meaning, guided by analytical interests. A broad coding frame, centred on the key topic areas addressed during the interviews—namely, (1) experiences of knowledge acquisition and skill enactment, (2) factors influencing skill enactment, and (3) perceived change in mental health—was used to address our research questions. Following data familiarisation, codes were developed and applied to the data under each topic by the first author (HMJ). Similar codes were grouped together to form initial themes using a primarily inductive approach. This approach was used to capture a broad range of participant experiences and perspectives, providing a useful foundation for understanding the outcomes studied. The themes were refined through review against the coded data extracts and interview transcripts. Themes are reported under each pre-determined topic area. Verbatim quotes are utilised to illustrate themes, although filler words such as “like” and “um” were removed for readability. Participants are identified using self-reported gender and a coded number (e.g., F1 = female, participant 1).

### Ethical considerations

All participants provided written informed consent after reading an information sheet in accordance with the Declaration of Helsinki. Electronic gift cards valued at $30 (AUD) were distributed to thank participants for their time. The study received ethical approval from the Australian National University Human Research Ethics Committee (protocol number 2023/561).

## Results

The themes and sub-themes are described within the broad topic areas targeted during the interviews and are presented in Table 2. We observed several contexts relevant to understanding experiences of skill enactment and knowledge acquisition among the participants. Firstly, participants were generally quite knowledgeable about mental health. About half of the participants had prior contact with mental health services either through their own treatment or by supporting a family member’s treatment, such as accessing services for a child or attending sessions as a partner. Others had used self-help resources in the past and a few had worked in or held qualifications in related fields. Secondly, this group demonstrated high program engagement, with all participants completing most, if not all, of their selected program. Participants had diverse motivations for using digital tools. Several cited the affordability of digital programs, while others preferred using digital tools or felt their concerns did not warrant more intensive support. Two participants signed up for a digital intervention due to barriers in accessing face-to-face services, with both beginning in-person treatment during their use of the digital intervention. Lastly, participants reported generally positive experiences with their selected intervention. Key benefits cited by participants included the provision of practical strategies to reduce distress, along with a general sense of being supported.

**Table 2:** Overview of topics, themes, and sub-themes

Topic/ Theme	Sub-themes
Topic 1: Knowledge acquisition and skill enactment	
Knowledge consolidation	i. Reinforcing existing knowledge ii. Enhanced comprehension and understanding
Active approach to skill enactment	i. Variability in skill selection
***Topic 2: Factors influencing skill enactment***	
Relevance of intervention strategies	i. User needs and characteristics ii. Lack of tailoring and customisation
Perceived and experienced benefit	–
Navigating time constraints and low motivation	–
***Topic 3: Impact on mental health***	
Perceived changes	i. Factors driving change

### Topic 1: knowledge acquisition and skill enactment

Two themes were developed to describe the data in relation to Topic 1: (1) knowledge consolidation (subthemes: (i) reinforcing existing knowledge and (ii) enhanced comprehension and insight) and (2) active approach to skill enactment (subtheme: (i) variability in skill selection). The first, knowledge consolidation, highlights how digital CBT programs enable users to revisit, strengthen, and build on their existing knowledge and skills related to mental health. The second theme, active approach to skill enactment, demonstrates how participants actively engage with intervention skills, both initially and over time. Differences were evident in the range and types of skills used among participants.

### Topic 1, theme 1: knowledge consolidation

#### Subtheme 1: reinforcing existing knowledge

Most participants highlighted that the information presented in their selected intervention was “*…not new*” (F10). Instead, it felt familiar or aligned with what they already knew or had encountered previously. For example, one participant remarked, “*there’s not necessarily a huge amount of novel information*” (M3), while another simply stated, “*I don’t think I learnt anything new at all*” (F8). Others commented that although they may not have explicitly known or articulated the concepts presented in the intervention, the information was not surprising because it was “*common sense*” (F1). Revisiting familiar concepts was generally viewed positively, with one calling the program a “*…good little refresher*” (F12) that helped reinforce their existing knowledge, and another stating, “*It doesn’t have to be rocket surgery. It’s just a reminder…*” (M3). This was important as some participants had forgotten things they previously knew, “*All these years. I was enough busy. I forgot all these things*” (F10). These reminders boosted participants’ confidence in their existing knowledge, with one participant commenting that it reminded them of some things they knew “*…and confirmed that that was a good coping strategy*” (F12). Others emphasised that the intervention reminded them to “*look out for*” (F5) themselves first. However, one participant whose mental health issues significantly impacted their daily life found these reminders distressing because they reminded them of their perceived inadequacies, “*I think maybe something to aspire to. Maybe somewhat more distressing though. Maybe just a reminder of how inadequate I am or how stuck I am*” (F8).

#### Subtheme 2: enhanced comprehension and insight

Most participants described gaining some knowledge or skills from their chosen intervention, although the degree of perceived learning appeared to vary based on their existing levels of knowledge. Many participants who were already quite knowledgeable pinpointed specific gains, “*ways to relax and… practice mindfulness… so there was some new things*” (M2). Conversely, those with limited knowledge emphasised broader insights, with two participants stating they “*learned a lot of things*” (F13, F5). Although participants were already familiar with some concepts, they also highlighted the “*…difference between having knowledge of something and then having that in an applicable form*” (M7). Many found that the accessible and structured format of the programs aided them in developing this knowledge, with one participant commenting that having the information and activities “*broken down into steps was really kind of helpful*” (F4), underscoring the value of this approach in fostering problem-solving skills:

*“…the whole problem-solving thing was really important. So*,* the problem solving*,* how to break things down into different steps*,* and how to approach things at a different level or slower.”* (F4).

In several cases, the interventions also led to a shift in the way the participants perceived and interpreted their emotions and experiences, “*the key takeaway… was that sometimes we really overthink our interactions with others*,* and that module really made me reflect on that… So*,* I keep that in mind even now*” (F1). Others described a shift in focus away from external factors beyond their control to their actions and internal thought processes:

*“A lot of it has to do with how we perceive and go about things. And so*,* you know*,* I guess the management side of it. We can manage it ourselves. So yeah*,* it was good to learn about that.”* (M2).

### Topic 1, theme 2: active approach to skill enactment

Participants generally demonstrated an active approach to applying the intervention skills, engaging with online activities initially and making concerted efforts to apply these strategies beyond their initial engagement. One participant stated, “*I’m trying to use those strategies. You know*,* I’m trying to sort of rewire my brain*” (F12, referring to cognitive restructuring). Another shared that they, “*… tried to practice the same things*” (M2, referring to lifestyle and sleep hygiene) after finishing their selected intervention, highlighting ongoing efforts to enact skills. Some participants also described developing personalised approaches to applying the skills, as highlighted by one participant when describing their efforts to achieve a balanced state of mind integrating both rational and emotional aspects:

*“So*,* it was sort of sort of as I was practicing wise mind. You know*,* within that unit sort of practicing it … And I was like*,* ‘Oh*,* yeah. I can talk to so and so. And I can talk to this person too. And they’re really good for sound boarding. Oh*,* yeah. So*,* I can use these people as wise mind soundboards’.”* (F6).

For some, implementing the strategies demanded less effort over time, with one participant stating that it had become “*really easy*” to think differently and commenting, “*…it is like a part of me now*” (F5). Others described it as a “*work in progress*” (F1) and challenges persisted for some, as indicated by one participant who remarked, “*as much as I know about sleep hygiene*,* implementing it has always been hard*” (F6). Sometimes, participants attributed these difficulties to their own capabilities or perceived shortcomings:

*“…sometimes I get really frustrated with my unhelpful thoughts and think well*,* ‘cause I’m 50*,* so it’s a bit different…to like a 20-year-old*,* where you’re able to kind of interrupt the patterns before it gets too ingrained. So sometimes I think…maybe I’m just too far gone.”* (F11, referring to cognitive restructuring).

Despite challenges, many participants intended to continue practicing the skills acquired from their selected intervention, with one noting, “*…obviously*,* I will just keep practicing it*” (F4, referring to problem-solving). Others had already revisited, or intended to revisit, the treatment materials in order to further develop their skills, “*I think it’d be good to probably revisit it…and go through*,* maybe do the challenge again*” (M2). However, one participant questioned how sustainable these changes would be over time, “*…at the moment*,* because it’s still quite recent and fairly fresh in my memory*,* I am quite aware of it*,* conscious of it. If that’s going to be sustainable long term*,* I don’t know*” (F1).

#### Subtheme 2: variability in skill selection

Although all participants engaged with the intervention skills, differences emerged in the number and types of skills used. Nearly half of the participants described trying most or all of the online activities initially, with one stating, “*I used everything*” (F5). However, others took a more selective approach, “*I tried some of them*” (M7). These differences persisted regarding skill enactment beyond participants’ initial engagement. Those with existing knowledge typically concentrated on integrating select skills. Many of these participants also described using various strategies to support their mental health prior to intervention, such as physical activity, hobbies, or social connections. Some among this group focused on integrating newly acquired skills, such as problem-solving techniques or fostering social connections, whereas others aimed to strengthen existing skills. Among those focused on improving existing skills, cognitive restructuring emerged as a prominent strategy, with one participant commenting, “*…managing thoughts is the key one for me*” (M3) and another stating that they were “*trying to do that a lot more*” (F12). Conversely, participants with limited knowledge or skill enactment prior to intervention generally adopted a broader range of strategies, emphasising a focus on establishing healthy habits and routines, “*I’m taking a lot of care of my diet…I started doing yoga. And I think it’s more like*,* peaceful for my mind. And kind of like connecting with nature*” (F13).

### Topic 2: factors influencing skill enactment

Three themes were developed in relation to Topic 2: (1) relevance of intervention strategies (subthemes: (i) user needs and characteristics and (ii) lack of tailoring and customisation), (2) perceived and experienced benefit, and (3) navigating time constraints and low motivation. The first theme, relevance of intervention strategies, underscores how the alignment of intervention strategies to the users’ unique needs and characteristics can facilitate or hinder skill enactment decisions. The second theme, perceived and experienced benefit, highlights how participants’ perceptions and experiences influence their judgments regarding the effectiveness of intervention strategies, which in turn shape initial and ongoing skill enactment. The final theme, navigating time constraints and competing priorities, emphasises the practical challenges participants encounter to integrating intervention skills into their daily routines amidst busy schedules and competing responsibilities.

### Topic 2, theme 1: relevance of intervention strategies

#### Subtheme 1: user needs and characteristics

All participants demonstrated an understanding of their unique symptoms and challenges. Recognising these challenges motivated participants to seek change and work towards personal growth and improvement, “*I always felt like I had this issue…and I thought that I should change…this bad habit. Obviously*,* it is kind of a habit*” (F5). Underlying participants’ motivation was a belief that these concerns could be addressed through psychotherapeutic intervention, with some preferring it over medication, “*…because the doctor kind of recommended me*,* okay*,* you should take antidepressants…but I wasn’t into pills or anything. I want to do it naturally*” (F5). The desire for change prompted participants to prioritise strategies that directly addressed their needs or concerns. Several focused on cognitive restructuring because it targeted their “*biggest problems*” (F12), while others integrated sleep hygiene and lifestyle practices or mindfulness, “*My thing is breathing*,* regulating my breathing*,* because that helps my heart rate*” (F6). Conversely, strategies perceived as less relevant were often disregarded, “*…I just didn’t wanna go right into that because I didn’t think that was relevant at the time*” (F4).

Participants’ decisions to enact skills were further influenced by the alignment between their perceived personal characteristics or abilities and the intervention strategies, as illustrated by one participant who found cognitive exercises suitable because they enjoyed introspective and analytical thinking: “*…I really like thinking about things. So*,* in kind of analysing my thoughts*,* why I might think a certain way…*” (F11). Less alignment reduced skill enactment, as illustrated by one participant who expressed doubts about their ability to maintain focus during mindful breathing practice:

*“…maybe my concentration and attention is not the best. And also*,* sometimes I kind of can get distracted very quickly*,* very easily. And that*,* I guess you need to have a lot of patience and be able to concentrate on that in order for it to work.”* (F1).

#### Subtheme 2: lack of tailoring and customisation

Several participants felt their selected intervention lacked tailoring to their individual needs, with some noting that this impacted their engagement with the online activities and motivation to apply skills. Some older participants noted that the interventions seemed to be “*…pitched to the younger age group*” (M3). Others highlighted a lack of tailoring to their current symptom levels or existing knowledge. For instance, one participant, who was experiencing severe mental health difficulties, felt overwhelmed by the amount of information provided in each module and suggested breaking it down over several weeks “*…to focus on week one*,* engaging in exercise*,* week two*,* engaging with food in a more kind of healthy way…rather than trying to look at the picture as a whole*” (F9). Another participant, a trained counsellor, observed, “*the course does seem to be tailored to people who maybe have never done CBT before*” (F11). Some participants commented that the lack of personalisation “*…kind of felt demotivating*” (M7), and several underscored the importance of providing tailored content to maintain engagement, “*it would be good to have a bit more tailoring or personalisation so you can kind of choose your focus areas. I think that would…enable me to feel more like I would keep using it on a more consistent basis*” (M2). Similarly, others suggested adapting content to the user’s existing levels of knowledge or skills, “*…maybe having something that’s geared towards somebody who’s had a lot of experience…maybe having that in the resources as well*” (F11).

### Topic 2, theme 2: perceived and experienced benefit

Participants’ initial evaluations of the potential value or benefit of a skill influenced enactment, with one participant stating, “*I’m always willing to give things a go when I can sort of see the value*” (F6) and another commenting, “*I did everything that I thought would be useful*” (F12). Others tried strategies despite initial scepticism as they were desperate to find relief from their symptoms:

*“At first… I’m not really believing in the progressive muscle relaxation*,* because I was thinking*,* what’s the point of tensing your body before you go to sleep…but because I was already frustrated*,* I’m trying it anyway. I’m thinking there’s no harm. I cannot sleep anyway.”* (F8).

Participants were motivated to persist with strategies that were experienced as helpful, whereas negative experiences reduced motivation. For example, one participant shared how encountering challenges with an online activity designed to identify cognitive distortions discouraged ongoing use:

*“…Like*,* there was this activity where it gave you a situation and you had to identify what kind of warped thinking it was. Was it catastrophizing? Was it this? Was it that? But that just annoyed me a bit. I felt I don’t need to be able to identify all those things.”* (F12).

Past experiences also informed participants’ judgements. Some favoured strategies that had been helpful in the past, while previous unsuccessful attempts deterred others from implementing similar strategies, “*I do remember*,* when I read some of the things*,* thinking*,* ‘I just can’t be bothered*,* this just doesn’t work’*” (F11). Concerns that engaging with certain strategies could lead to negative emotional consequences also influenced strategy selection among some participants, with one questioning whether engaging with certain strategies would “*have brought up other things*” (F4).

### Topic 2, theme 3: navigating time constraints and low motivation

Difficulty integrating the strategies into everyday life due to time constraints was mentioned by many participants as a barrier to both initial and sustained skill enactment. Busy work schedules and domestic responsibilities were both highlighted as getting in the way of consistently implementing skills. One participant succinctly summarised this challenge, “*You know*,* it’s just life…there’s too many things going on for you to do all those things and have that same routine every single day.”* (M2). This struggle was particularly relevant when implementing time-consuming strategies like exercise, though it was less relevant to strategies that were easily integrated into existing routines, such as those that “*… didn’t involve having to move or go somewhere*” (M7) or “*didn’t sort of take too much…time*” (M2). One participant suggested that prioritising other tasks over time-consuming and effortful activities like exercise was perhaps “*…a way of me subconsciously trying to get out of it*” (F11), suggesting issues related not just to lack of time but also motivation and prioritisation. Difficulties with motivation were often described as an ongoing challenge requiring perseverance and self-discipline, as expressed by one participant:

*“…I find it quite draining to just keep tapping into that parental aspect of my psyche that says*,* ‘Come on*,* you know what you need to do’. But just the child part of me goes*,* ‘No*,* I’m sitting here watching my favourite show. Go away’.”* (M3).

Participants described various techniques to overcome challenges due to lack of time or motivation. Some embraced a “*just do it*” (F6) attitude, while others described using cognitive strategies to remind themselves of the benefits of action or the consequences of inaction:

*“If I have an unhelpful thought*,* like*,* for example*,* ‘I can’t be bothered going on my bike ride’. Then thinking about…the consequences of not doing it*,* and then thinking about alternative ways I can think of it…like*,* instead of ‘I don’t want to go for a bike ride*,* I’m too tired.’ Think*,* ‘Well*,* yesterday*,* I went for a bike ride. I was tired*,* but I made myself do it. When I came home*,* I felt much better’.”* (F11).

One participant described actively planning actions into their daily routine to facilitate ongoing engagement: “…*two days I do yoga and two days I do gym workout…and two days I go for swimming. So*,* I’ve kind of divided the time*” (F13). Reminders to engage with the intervention skills were also recommended by some participants because “*sometimes you listen to something and you totally forget*” (F13). Another participant suggested that online programs could incorporate “*time sensitive prompts*” (F8) to remind users to engage with adaptive actions when they are most needed.

### Topic 3: changes in mental health and wellbeing

One theme was developed in relation to topic 3: (1) perceived changes (subtheme: (i) factors driving positive change). Participants generally perceived some positive change in their mental health associated with use of the programs and enacting the skills they learned, although this was typically modest. Key factors contributing to change varied across participants but included factors related to improvements in skill enactment and knowledge acquisition.

### Topic 3, theme 1: perceived changes

Most participants described experiencing some positive changes in their mental health or wellbeing as a result of using their selected intervention. The changes encompassed reductions in anxiety or worry, improved mood and relationships, better sleep quality, increased emotional resilience, and improved coping. For many, these changes were modest, with one participant commenting, “*…maybe I’m a little less worried*” (F1) and another stating, “*I think I can sleep easier*” (F8), despite some ongoing difficulties. However, a couple of participants described more substantial changes, with one remarking, “*…it kind of changed my whole life*” (F5), while another stated, “*I feel great. I feel better than before*” (F13). Notably, two participants, both of whom were seeking more intensive support, noted a decline in their mental health. In both cases, this was attributed to external factors rather than the intervention (e.g., difficulty scheduling regular appointments with a new therapist).

#### Subtheme 1: factors driving positive change

Participants identified several factors contributing to the described improvements. Common across most accounts was the importance of applying the strategies presented in everyday life. Several participants emphasised the need for ongoing practice and “*sticking to routines*” (M2), recognising that consistency in implementing skills played an important role in their improvement. For instance, one participant commented, “*My biggest takeaway from all of it was that I need to keep doing it. It’s not something that you just do every now and again*,* or you just do when your health flares…it’s a maintenance job…*” (F6). Improvements in knowledge and understanding were also highlighted, with some noting that new insights led to a shift in their perspective or approach to situations, such as “…*about how you think in social situations about maybe not*,* kind of taking a step back and not overthinking*” (F1). Several participants underscored the importance of these factors alongside other elements such as engaging supportive social networks and a commitment to growth and change, as one participant commented:

*“But the most important thing is…if I am ready*,* then everything will work out. But if I’m not ready*,* I’m doing it forcefully…I will not be happy and there will be no change. Nothing can work out if you don’t want to.”* (F10).

Together, several participants described how these factors contributed to an enhanced feeling of control and empowerment through participation in these programs. For instance, one participant remarked, “*I think my anxiety has sort of calmed down*,* because…when I think okay*,* so this is why it might be happening*,* and this is what I can do. I think it’s down*” (F4), while another commented, “*I feel like I am more in control of my situation and how I respond to it*” (F6). Additionally, two participants highlighted the importance of human support or guidance, with one emphasising the value of shared experience, “*…she [the therapist] moved to Australia*,* maybe 15 years ago*,* so she knows what it’s like to kind of move countries and yes*,* so*,* I found that really helpful*” (F11).

## Discussion

This study explored the perspectives and experiences of adults utilising publicly available digital CBT programs in Australia, with a focus on skill enactment and knowledge acquisition. The study aimed to understand how community users learn and apply knowledge and skills, identify factors influencing skill enactment, and explore perceived changes in mental health. Thirteen adults who had utilised 13 interventions from eight different service providers participated. All of the participants demonstrated high program usage. Overall, the findings suggest that DMHIs can reinforce existing knowledge, facilitate the acquisition of new insights, and encourage a proactive approach to implementing practical strategies in everyday life, particularly among users who actively engage with these interventions.

Participants emphasised that revisiting familiar concepts and information in these interventions helped reinforce their existing knowledge and improve their confidence to manage their mental health. This is in line with previous trials of DMHIs, which have indicated improvements in knowledge confidence following intervention [43, 44]. In the area of health education, repetition of health messages is considered crucial to improve retention and knowledge confidence [45], especially considering patient recall of health information is frequently poor [46]. These insights underscore the importance of ongoing education and reinforcement in promoting effective self-management and self-efficacy among users. The finding that these interventions offered participants new skills and insights is generally consistent with a study by Berg et al. [28], which found that adolescents who actively engaged with a digital CBT intervention for depression gained new perspectives and strategies to manage their mental health. Our study also extends on these findings by highlighting variability in the degree of learning based on pre-existing knowledge: participants with less prior knowledge tended to emphasise substantial gains, while those with more knowledge typically reported specific improvements in particular areas or skills.

Overall, the findings highlight that participants generally adopt an active approach to applying intervention skills, evident in their engagement with online activities and ongoing efforts beyond the initial intervention period. Similar to the findings for knowledge acquisition, differences also emerged in the number and range of skills enacted, with some participants integrating a broad range of skills into their routines while others focused on specific strategies. Research on emotion regulation highlights different aspects of skill enactment that may be relevant for outcomes, including the range or breadth of skills employed (i.e., quantity), how often skills are used (i.e., frequency), and the proficiency with which skills are implemented (i.e., quality or fidelity) [47]. Results of this study suggest that a user’s baseline level of knowledge or skills use may play a key role in the impact of interventions on skill enactment. Individuals who already possess a diverse repertoire of skills before intervention may prioritise mastering a smaller subset of skills, whereas those with fewer skills may concentrate on learning and exploring a broad range of skills, identifying skills that work best for them, or establishing healthy routines.

Our finding that participants generally adopted an active approach to learning and applying skills differs from results reported in previous qualitative studies conducted in trial settings [27, 28]. These earlier studies identified a subset of users exhibiting a more passive approach to learning and engaging with skills, despite also interviewing participants with relatively high levels of program engagement. Methodological differences, such as the use of trial procedures, variations in sampling strategies, and the timing of interviews, may account for these differences. For instance, Halmetoja et al. [27] used a criterion-based sampling approach based on the degree of symptom improvement, and both previous studies interviewed participants 6 months to 4 years post-intervention. Similar to these studies, our study also does not capture the experiences of the majority of individuals who drop out of digital interventions early [11]. Our recruitment method primarily attracted highly engaged, motivated, and relatively knowledgeable participants. Experiences of skill enactment among this group are likely to differ from those who have limited previous experience with mental health, less readiness or motivation to change, or a more passive approach to learning. Future studies should recruit specifically for people facing challenges in engaging with and using information from these interventions, such as those who report a poor user experience or have low levels of mental health literacy.

Decisions to enact certain strategies were influenced by their perceived alignment with participants’ mental health needs and personal characteristics, as well as their perceived or experienced effectiveness. This is similar to a previous study by Eilert et al. [48], which found that in a pragmatic randomised controlled trial of internet-based CBT for anxiety and/or depression, the selection of skills was partly driven by perceived symptoms and strategy effectiveness. These findings are in line with the common-sense model of self-regulation [49], suggesting that the selection and maintenance of skills is guided by an individual’s cognitive representations of their mental health concerns and beliefs about how effective different interventions will be in addressing those concerns. The findings suggest the importance of considering the beliefs, experiences, and goals of users when designing, targeting, or tailoring intervention programs. Failure to personalise interventions to these experiences may reduce the feeling of being personally connected to the intervention and diminish participants’ motivation to engage, as seen in this study. Personalisation of digital interventions can be achieved through user or provider choice, rule-based algorithms (e.g., presenting content relevant to clinical symptoms), and data-driven approaches that tailor therapeutic recommendations based on a user’s previous interactions and behaviour within an intervention [23, 50]. However, there is currently a lack of published research investigating these approaches based on user knowledge and skills.

Time constraints due to paid employment and domestic responsibilities were emphasised as barriers to the maintenance of skills. These factors were especially pronounced for time-consuming strategies like physical activity, whereas activities that were easy to implement in everyday life presented fewer barriers. Research consistently identifies lack of time as a key barrier to both engagement with DMHIs [12] and health-related behaviours such as exercise participation [51]. However, recent studies suggest that perceived time scarcity may obscure more important barriers, such as difficulties with prioritisation or motivation [52]. It was evident that sustaining motivation could be challenging for participants. Maintaining health actions over time involves recognising their importance for mental health and wellbeing and choosing to prioritise them over other activities or commitments that may be less essential. Consistent with existing behavioural theories and evidence-based behaviour change techniques [53, 54], the participants observed that this process could be facilitated by various factors, including adopting a “just do it” attitude, creating action plans, and reminding themselves of the benefits of engaging in mentally healthy actions. These frameworks also recommend additional strategies not explicitly mentioned by the participants, such as goal setting, leveraging social support features, and implementing rewards systems, to sustain adaptive behaviours over time [53].

The findings show that most participants perceived positive changes in their mental health and wellbeing because of the intervention, including reduced anxiety, improved mood, better sleep quality, and enhanced relationships. Although many described these changes as modest, some participants reported substantial improvements. Importantly, two participants who were seeking more intensive support noted a decline in their mental health. This was attributed to external factors but could suggest that existing programs may not be sufficient for individuals with higher levels of need. Understanding the factors driving change in psychotherapies is crucial for developing more effective intervention packages, but evidence on causal factors remains largely uncertain [55]. Participants highlighted various factors as relevant to the observed improvements, including the consistent application of skills, experiences of learning, and a commitment to personal development and growth. Together, these factors contributed to a sense of empowerment and control among the participants over their mental health and wellbeing. The findings are consistent with recent research on the mechanisms of change in DMHIs [56], and suggest the importance of active coping and self-management in intervention effectiveness. These concepts extend beyond skill enactment to capture a wide range of activities undertaken to actively manage mental health and wellbeing, including not just the implementation of specific strategies but also factors such as adherence to treatment protocols, social support, and symptom monitoring. Continued research into mechanisms underlying these interventions is essential for program optimisation and addressing the diverse needs of individuals seeking support through digital tools.

### Implications

This study provides valuable insights for future research and intervention design. Although participants generally responded positively to the interventions, the findings also suggest the importance of personalised content that targets the primary concerns of users and is relevant to their existing levels of knowledge and skills. Individual differences in existing knowledge and skills suggest a need for separate program paths that include both introductory CBT programs offering step-by-step guidance, as well as more advanced programs that assume a level of knowledge based on previous intervention or ongoing face-to-face services. Providing options for users experiencing high levels of need or chronic impairment—such as relapse prevention programs or adapting models of chronic illness management—may also be needed given that these challenges were observed among some participants in our study. Future studies could also explore the effectiveness of advanced personalisation techniques such as adaptive learning models that dynamically adjust therapeutic content recommendations and feedback based on analysis of the user’s previous interactions within the intervention. Concerns about the sustainability and long-term maintenance of skills underscores the need to explore effective approaches for providing ongoing support and resources (e.g., periodic booster sessions, self-monitoring tools, and peer support networks) to support skill enactment beyond the intervention period. Additionally, findings related to knowledge acquisition suggest the importance of integrating learning support strategies and offering opportunities for ongoing education to support knowledge retention and confidence. Future trials are warranted to examine the relevance of key constructs identified in this study, such as knowledge confidence, practical knowledge, and active coping, for mental health outcomes.

### Limitations

This study has several limitations. Data were collected from a sample of participants who self-selected into the study. The participants generally demonstrated high levels of insight, several had previous experience with mental health services, and most described predominantly positive views of their selected intervention. Consequently, the findings may be biased towards individuals who are relatively knowledgeable and have had positive experiences with these interventions. Results might differ among a more naïve sample of users or those who have negative experiences. Additionally, although the sample varied in demographic characteristics such as age and self-reported ethnicity, most participants were female, and there was limited representation of participants with lower levels of education. Males tend to have lower rates of mental health service utilisation than females [57], and those with lower levels of education often face challenges with mental health literacy [58] and overall health outcomes. Future studies should prioritise the inclusion of these underrepresented groups using strategies such as maximum variation sampling to recruit participants with different levels of mental health knowledge and varying demographic characteristics [59]. Personalised recruitment invitations (e.g., targeting those with negative user experiences) and offering different options for participation (e.g., offering interviews via email or other online platforms) may help ensure representation among individuals who are less likely to engage.

In addition, many of the interventions used by participants in this study were only available to users in Australia [60], most had government funding, and all were accessible at low or no cost to users at the time of study. The availability and characteristics of digital interventions are likely to vary across countries due to differences in healthcare systems, cultural contexts, and support structures. These factors may influence how users engage with and benefit from DMHIs, suggesting the need for further research to explore these questions across different geographical contexts. Insights from this study are further limited to exploring relatively short-term effects on knowledge acquisition and skill enactment based on participant self-report. Studies conducted over longer time frames are needed to understand how skill enactment evolves over time and influences longer-term mental health outcomes. Participants may also not have complete insight into their motivations or the mechanisms through which interventions improve outcomes. Supplementing the results with ecological momentary assessment studies could help better understand temporal relationships between motivations, program use, skill enactment, and health outcomes. Moreover, although our study considered the impact of pre-existing knowledge on the outcomes studied, it did not explicitly investigate the influence of current or previous engagement in treatment services. Additionally, we did not explore in detail the specific features or components of digital interventions that might contribute to learning and skill enactment (e.g., interactive features or feedback mechanisms). Future studies exploring how treatment histories and the features of digital interventions may affect engagement with and responses to digital interventions are warranted. Future studies should seek feedback from participants on study findings to ensure that their unique meanings and perspectives are accurately represented and not constrained by the researchers’ own knowledge or goals.

## Conclusions

Better understanding of skill enactment and knowledge acquisition can help foster innovation in the design and implementation of DMHIs. This study addresses an important gap in the literature by employing qualitative methods to explore skill enactment and knowledge acquisition with a sample of community users. Among a group of engaged and motivated users, our findings suggest that DMHIs are valuable, in part, because they help reinforce and strengthen existing knowledge and confidence, offer new insights and skills, and encourage the proactive application of skills. Variability among participants in their existing levels of knowledge, skills, and preferences for therapeutic strategies underscores the need for adaptive interventions based on user choice or machine learning algorithms that dynamically tailor therapeutic content based on user behaviours within an intervention. Future research should focus on establishing effective approaches for personalising DMHIs, and explore how to best support the acquisition, retention, and sustained use of knowledge and skills.

### Electronic supplementary material

Below is the link to the electronic supplementary material.

Supplementary Material 1

## Data Availability

The datasets generated and/or analysed during the current study are not publicly available due to their sensitive nature but deidentified data are available from the corresponding author on reasonable request.

## Abbreviations

CBT: Cognitive behavioural therapy
DMHI: Digital mental health intervention
RCT: Randomised controlled trial
COREQ: Consolidated Criteria for Reporting Qualitative Research
